# An Examination of the Effect of Yogurt Consumption on Nutrient Quality of the Diets of Canadians Across the Ages

**DOI:** 10.3390/nu18101581

**Published:** 2026-05-15

**Authors:** Hrvoje Fabek, Mavra Ahmed, Sylvie S. L. Leung Yinko, Peggy Drouillet-Pinard, G. Harvey Anderson

**Affiliations:** 1 Department of Food Science, University of Guelph, Guelph, ON N1G 2W1, Canada; 2 Department of Nutritional Sciences, University of Toronto, Toronto, ON M5S 1A8, Canada; 3 Joannah and Brian Lawson Centre for Child Nutrition, University of Toronto, Toronto, ON M5S 1A8, Canada; 4 Danone Canada, Boucherville, QC J4B 1E6, Canada; 5 Danone Global Research and Innovation Center, 91190 Gif-sur-Yvette, France

**Keywords:** dairy, yogurt, matrix, nutrient quality, protein, calcium, Canadian Community Health Survey, children, adults, elderly

## Abstract

Background/Objectives: Dairy yogurts are a source of protein and micronutrients in the Canadian diet. However, Canada’s Food Guide emphasizes the consumption of plant-based foods, which is facilitated by a greater availability of dairy alternatives on the market. The nutritional composition of these products varies and can differ from dairy foods such as yogurt, which contain high-quality protein and micronutrients. The objective of this study was to examine the effect of dairy yogurt consumption as part of a diet on any given day on nutrient intakes in Canadians across ages. Methods: The 2015 Canadian Community Health Survey (CCHS)—Nutrition first day 24 h recalls of males and females > 1 years of age (n = 17,308) and of yogurt consumers (n = 3788) were examined to estimate nutrient intakes arising from yogurt consumption. Respondents were allocated into four groups defined by their daily yogurt intake in grams (i.e., Group I/non-yogurt consumers: <1 g; Group II: 1–90 g; Group III: 90–115 g; Group IV: >115 g). Results/Conclusions: The results of this study provide timely data on Canadian yogurt consumption across the ages and show that those consuming yogurt have higher intakes of essential nutrients, such as protein, calcium, potassium, vitamin D, and dietary fibre. The data from this study emphasize the importance of yogurt in the context of a healthy eating pattern and emphasize the need to encourage consumption of yogurt within Canada’s Healthy Eating Strategy.

## 1. Introduction

Dairy consumption in Canadians has decreased in the last 30 years, where dairy products are commonly being replaced by a preference for dairy alternatives [1]. On the other hand, rates of overweight, obesity and Type 2 Diabetes (T2D) in Canada continue to increase. We have recently reported on the impact that varying the animal/plant protein ratio has on nutrient and protein quality in Canadians across the ages [2,3]. However, understanding how dairy foods impact nutrient intakes across different life stages in Canadians has not been assessed. A recent study showed that consumption of milk and alternatives in adults (>19 years of age) provided a range of essential nutrients but that they failed to meet the recommendations for milk and alternatives outlined in the 2007 Canada’s Food Guide (CFG) and that dairy intakes decreased with age [4]. It was also recently reported that yogurt should be encouraged in healthy eating patterns due to its high nutritional value, flexible food matrix, beneficial bacterial components, and familiarity amongst consumers [5]. However, the types of dairy foods consumed and how and when they are consumed among Canadians from children to elderly remain unclear. This research will fill this knowledge gap.

As dietary requirements differ by age, we aim to examine dairy consumption at different life stages (children, pre-teens, teens, adults, and elderly) as well as evaluate the pattern of consumption of dairy (snacks vs. meals). Data from the Canadian Community Health Survey (CCHS-2015) were used to model dairy intakes by age groupings. This is timely as Health Canada’s Guiding Principles and WHO recommendations for dietary guidance encourage consumption of plant-based meals and plant protein sources to counter current disease trends. However, our recent reports demonstrate that Canadians consuming >50% plant protein on any given day are at risk of under-consuming protein, vitamin B12, calcium, vitamin D, among other nutrients [3]. As a result, a de-emphasis of milk and dairy foods in the current CFG may have unintended consequences in terms of nutrient adequacy, particularly if Canadians replace dairy with less nutrient-dense alternatives. Plant alternatives to dairy are widely available on the market but are lower in protein quality, lack the nutrient density of dairy and most importantly do not contain the unique food matrices of different dairy foods such as fluid milk, cheese, and yogurt [6].

Milk and dairy products are a source of protein, vitamin A, vitamin D, vitamin B12, and riboflavin as well as calcium, phosphorus, magnesium, and potassium [7], and, for this reason, they have been a part of the CFG since the 1940s. However, the new CFG 2019 has eliminated the “Milk & Alternatives” food group and instead is emphasizing lower-fat dairy products as a subgroup of protein foods [8]. This major change in the new CFG may not only affect the intake of yogurt and dairy products in the Canadian population but, more importantly, the intake of essential and non-essential nutrients. This is vital in the context of healthy aging for both growing children and teens as well as the elderly. For the latter, it has been suggested that high-quality animal proteins in the diet, such as dairy, should be maintained to ensure protein adequacy, and this is considered a valuable dietary strategy for elderly individuals to avoid loss of muscle mass [9]. Understanding the dairy consumption patterns of Canadians (type, amount, and at what meal occasion) and their effects on nutrient intakes as a measure of diet quality of eating patterns will be informative for public health and provide timely data for food innovation strategies for plant-based alternatives. However, to our knowledge, there are no investigations on the consumption of dairy products such as yogurt and their effect on overall diet quality in Canadians across different ages.

The objective of this study was to examine and compare nutrient intakes reported by Canadians across the ages within groups of increasing yogurt consumption as part of their diet on any given day. We hypothesized that greater consumption of yogurt will result in improved nutrient intakes, including protein along with a range of micronutrients.

## 2. Methods

### 2.1. Data

Data from the CCHS 2015 Public-Use Microdata File (PUMF) were used for this study. Detailed information on the sampling of respondents can be found in the CCHS, Nutrition user-guide [10]. The CCHS 2015 is a voluntary, nationally representative survey conducted by Statistics Canada. This survey has a cross-sectional design with 3 sampling stages. Interviews were computer-assisted using a 5-step automated multi-pass method adapted and modified for the Canadian population from the United States Department of Agriculture and were conducted between 2 January 2015 and 31 December 2015, on all days of the week. Two separate questionnaires, (a) one 24 h dietary recall and (b) a detailed support document to complement the 24 h dietary recall, were administered. This information collected by Statistics Canada provides the most current nutrition data available for Canadians. The survey is representative of 12 age–sex groups that correspond to the Dietary Reference Intakes (DRI) groupings [11]. Final sample of the CCHS included 20,483 respondents, with a 61.6% response rate. During the first interview, a random subset of 35% of initial respondents were asked to complete a second 24 h dietary recall over the phone within 3–10 days of the first interview; however, for this study, only the one-day 24 h recalls were used.

### 2.2. Participants and Data Collection

Surveyed participants for CCHS 2015 were ≥1 year of age residing in Canada’s 10 provinces, and excluded individuals were living in territories, on reserves, Aboriginal settlements, full-time members of the Canadian Armed Forces, and institutionalized individuals. Recalls from breastfeeding women and individuals with invalid dietary recalls (as defined by Statistics Canada) were removed. CCHS 2015 PUMF does not identify pregnant participants. For this report, the single 24 h dietary recalls of males and females across all ages were utilized. The final sample was n = 17,308 (9372 adults, 2445 elderly and 3595 children).

### 2.3. Adjusting for Misreporting

To account for over- and under-reporting of energy intake, total energy expenditure (TEE) for each respondent was calculated based on the method reported by Garriguet et al. [12]. Reported energy intakes (EIs) were compared with each respondent’s TEE to identify mis-reporters. Under- and over-reporting was defined as the ratio of EI:TEE < 0.7 and >1.42, respectively, whereas those in between were considered plausible reporters. Briefly, the Institute of Medicine equations were used to predict TEE based on availability of age, sex, height, weight, and physical activity levels (i.e., sedentary, low active, moderately active and highly active) when measured height and weight or self-reported height and weight were available in the dataset. Adults’ (19 years of age and older) self-reported height and weights were adjusted using a correction factor applied by Statistics Canada [13].

Body mass index (BMI) categories were defined according to Health Canada’s guidelines [11]. To categorize physical activity level in 2015, respondents’ average physical activity per day in minutes was computed by dividing the variable PHSGAPA (hours of physical activity per week) by 7 and multiplying by 60. Cut-offs to define sedentary, low active, active, and very active were then applied from Health Canada’s Reference Guide to Understanding and Using the Data for CCHS 2015 [10], where underweight respondents (19 years of age and older with a BMI < 18.5 kg/m^2^) were excluded. Analysis was carried out on all plausible reporters (n = 17,308).

### 2.4. Definition of Yogurt Consumption Groups

Respondents were allocated into 4 groups defined by their yogurt intake in grams (i.e., Group I/non-yogurt consumers: <1 g; yogurt consumers—Group II: 1–90 g; Group III: >90–115 g; Group IV: >115 g), based on the single serving reference amount of yogurt as 115 g [14]. The “Bureau of Nutritional Sciences (BNS) Food Group Codes and Descriptions 2015 Canadian Community Health Survey-Nutrition” file was used to classify types of yogurt consumed by each respondent. Each type of yogurt is identified by the FID_CDE code in the FID file of CCHS.

### 2.5. Nutrient Intakes for Yogurt Consumption Groups

Nutrient intakes, macro- and micronutrient amounts (g or μg/day), were assessed within each yogurt consumption group, as previously in adults and elderly [2], as well as in children [15]. Group III was selected as the statistical comparator as it includes the commercial serving sizes and Health Canada’s reference amount of yogurt [14]. Although the survey data are usually presented for all ages years, we examined the nutrient intakes in the oldest age (70 years) group separately because sarcopenia and general malnutrition are of increasing concern in elderly populations [16].

### 2.6. Statistical Analyses

Balanced Repeated Replication with 500 replicates, as recommended by Statistics Canada, was used to estimate standard errors [17]. Survey weights were used to ensure samples from CCHS 2015 remain nationally representative. Data preparation and statistical analyses were completed using R Studio version 1.2.5019.

Data for macro- and selected micronutrients were expressed as amounts per 1000 kcal. Analysis of variance (ANOVA) by SVYGLM with LSMEANS function was performed to analyse nutrient intakes between the 4 groups. A multiple-comparison procedure for comparisons with a control was used to compare the reference group (90–115 g) with the other 3 groups. The significance level was set at *p* < 0.05 for significant differences with the reference group.

## 3. Results

The demographics and final sample size of each of the yogurt consumption groups are reported in Table 1.

For the yogurt consumers (n = 3788), the average amount of yogurt consumed daily was 131 ± 3.07 g. Yogurt consumed (in grams) by children, adults and the elderly was 123.78 ± 3.69, 135.51 ± 4.27 and 119.96 ± 4.31, respectively. The amount of nutrients coming from yogurt is presented in Table 2.

The highest amount of yogurt consumed was in females, 19 to 30 years old, followed by males, 31 to 50 years old (Table 3). We have also reported on the nutrients consumed from the yogurts only, excluding other food sources (Table S1).

Most yogurt was consumed as a snack (54.91 ± 2.37 g), followed by breakfast (39.06 ± 2.10 g) and lunch (29.59 ± 2.76 g). Although similar trends were observed for the adults, in the elderly, most yogurt was consumed at breakfast (37.92 ± 3.51 g), followed by as a snack (33.95 ± 4.43 g), whereas for children, most yogurt was consumed as a snack (59.41 ± 3.46 g), followed by brunch (48.25 ± 20.41 g), where breakfast (27.59 ± 2.14 g) and lunch (26.29 ± 2.09 g) intake was similar.

Yogurts 2 to 6% flavoured were the most consumed types of yogurts among all age groups, followed by yogurt beverage by toddlers and children. In adults and the elderly, yogurt, 2 to 6% plain, was commonly consumed.

### Effects on Nutrient Intake

When nutrient intakes relative to energy intake across groups of yogurt consumption were compared with the average comparator group (group 3, >90 g to 115 g) (Table 4), respondents in the first group (<1 g) had lower intakes of carbohydrates, total sugars, fibre, total fat, calcium, vitamin D and potassium, with high intakes of sodium (*p* < 0.05). Similarly, respondents in the second group (1 g to 90 g) had lower intakes of total sugars, calcium, and vitamin D (*p* < 0.05). Increased yogurt consumption (>115 g) resulted in higher intakes of energy, protein, saturated fat and calcium and lower intakes of sodium (*p* < 0.05). On average, yogurt contributed 6% (group 2), 11% (group 3) and 23% (group 4) total protein.

Results were similar for some nutrients by age groups (Table 5, Table 6 and Table 7). For example, for adults, respondents in the first and second groups (<1 g, 1 g to 90 g) had lower intakes of total sugars, calcium, and potassium, while respondents in the fourth group (>115 g) had higher intakes of protein but lower intakes of sodium *p* < 0.05). For children, the findings were also similar to the first group, having lower amounts of total sugars, protein, calcium, vitamin D and potassium but higher amounts of sodium. One of the differences was the increased total and added sugars for the fourth group (>115 g) (*p* < 0.05).

## 4. Discussion

In the present study, using nationally representative data from the CCHS-2015, we aimed to report how consumption of yogurt products across the ages (≥ 2 years) among the Canadian population affects nutrient intakes. On average, the mean intake of yogurt (g/d) in children, adults, and the elderly was 123.78 ± 3.69, 135.51 ± 4.27 and 119.96 ± 4.31, respectively. These trends are similar to a recent study analysing the NHANES database, which found that the mean intake of yogurt among US consumers was 150 ± 3 g/d and 182 ± 3 g/d among children and adults, respectively, which was, on average, approximately 1 serving per day. The authors concluded that regular yogurt consumption contributes to meeting the recommendations for dairy foods set by the Dietary Guidelines for Americans [18]. Despite the CFG having moved away from recommending serving sizes to Canadians, our results support the regular consumption of yogurt in a daily dietary pattern.

In the present study, yogurt consumers had significantly higher intakes of protein, calcium, potassium, vitamin D, and dietary fibre and lower intakes of sodium than non-consumers. On the other hand, energy and total fat did not differ between consumers and non-consumers across all three age groups, which was also reported by Vatanparast et al. [19]. Similar observations were also made in both US and international cohorts, where greater yogurt consumption resulted in improved intakes of these nutrients [18,20,21,22]. Many of these nutrients are currently under-consumed by Canadians, where calcium, potassium, and vitamin D have been identified as micronutrients to encourage due to inadequate intake and, therefore, must be declared on the Nutrition Facts Table on most prepackaged foods [23]. Moreover, it was recently reported that the absence of dairy consumption, particularly yogurt, would make it difficult for many individuals to meet some nutrient requirements [7]. The 2019 CFG has moved away from the concept of food groups in place of a snapshot illustrated by a plate containing vegetables and fruits, whole grains, and variable protein foods. Specifically, the current CFG emphasizes consuming plant proteins more often. According to Health Canada, many Canadians were not meeting the recommendations of the 2007 CFG and, as a result, the prevalence of nutrient inadequacy for micronutrients such as calcium, zinc, and vitamin A was high [24]. Dairy foods are a rich source of nutrients, and a deemphasis of dairy within the current dietary guidelines may have unintended consequences in terms of nutrient adequacy for Canadians as well as the health benefits dairy foods can provide.

The 2019 CFG encourages greater consumption of whole grains along with nuts, seeds, and legumes [8]. However, in our earlier analysis of protein intakes in Canadians, dairy foods remained as a top three preferred protein source in children, adults and the elderly [2,15]. The current CFG has eliminated its previous recommendations for frequent consumption of dairy, and they fail to be recognized on the CFG plate. Despite being identified as a protein source in the Healthy Eating Strategy, the emphasis is on low-fat dairy products, which most often compromise fat for refined starches and sugars. Health concerns related to dairy consumption have focused on its saturated fat content and its association with cardiovascular disease [25]. In contrast, recent research suggests that dairy saturated fat does not contribute to that association. A 12-year follow-up within a male cohort found high-fat dairy inversely associated with central obesity development compared to low-fat dairy [26]. In addition, a 12-week randomized, double-blinded interventional study in 52 adults with abdominal obesity found that a higher intake of dairy saturated fatty acids led to an increase in lean body mass and reduced total body fat percentage [27]. Furthermore, a systematic review and meta-analysis revealed that childhood obesity and overweight are 38% less likely to occur for high-dairy-intake patterns compared to low-dairy intake [28]. Moreover, a recent report demonstrated that total and full-fat dairy but not low-fat dairy is inversely associated with markers of developing metabolic syndrome [29]. These studies suggest that public health initiatives should move away from focusing on dairy as a source of saturated fats and should instead recognize the nutrient density of dairy products and their role in promoting bone and cardiometabolic health [30,31,32].

Most Canadians do not have adequate intake of dietary fibre, with the majority consuming less than half of the recommended amount [33]. In our study, non-yogurt consumers had significantly lower overall fibre intakes (Table 1). Since most yogurts on the market are not sources of fibre, these results suggest that yogurt is not typically consumed in isolation and, instead, is commonly consumed along with other fibre-rich foods such as nuts, fruit, and whole grain granola. We have previously shown that dairy beverages (milk and yogurt) consumed with a high glycaemic cereal at breakfast decreased food intake and increased satiety compared with water with cereal [34]. Our results support the need for similar research to be carried out in yogurt products as yogurt consumption has been associated with better diet quality [19]. Similar findings were observed in a US cohort, where both children and adult yogurt consumers reported better diet quality scores, assessed by HEI-205, than non-consumers. The authors observed that intakes of dairy, fruits, vegetables, and whole grains were significantly higher in yogurt consumers than non-consumers [18].

In addition to improved dietary fibre intakes, we also observed reduced sodium intakes among the yogurt consumers. Excess sodium intake is a major public health concern due to its association with hypertension and cardiovascular disease. According to Health Canada, many Canadians exceed the recommended sodium intake, with processed and packaged foods being the primary contributors [35]. Yogurt, particularly plain and unsweetened varieties, is naturally low in sodium, making it a beneficial addition to diets aimed at reducing sodium levels. The observed lower sodium intakes among yogurt consumers may reflect dietary patterns that favour whole, minimally processed foods [18]. Yogurt is often paired with nutrient-dense options such as fruits, nuts, and seeds, which contribute to a healthier overall diet. These dietary patterns are consistent with the emphasis in the CFG on whole and minimally processed foods. However, the shift away from explicitly promoting dairy in the guide raises questions about whether individuals may inadvertently miss out on the benefits of dairy products like yogurt, including their potential role in moderating sodium intake.

Frequent consumption of added sugars from foods has been associated with an increased risk of developing obesity and T2D [18]. Although Health Canada does not have explicit recommendations for added sugars beyond limiting them in the daily diet, Heart & Stroke recommends consuming no more than 10% total calories per day from added sugars [36], similar to recommendations by the Dietary Guidelines for Americans [37]. In our study, respondents across the ages reported to be consuming less than the recommended amount, even in the fourth group where yogurt consumption was greatest. A recent study showed that flavoured yogurts are popular in middle- and high-income countries and that most contain high concentrations of both total and free sugar. The authors concluded that regular consumption of flavoured yogurts could significantly increase total sugar intakes [38]. In our analysis, 2 to 6% flavoured yogurt was the most consumed type among all age groups. However, in the present study, the intake of added sugars was similar between non-consumers and consumers in children, adults, and the elderly (Table 3, Table 4 and Table 5), indicating that yogurt consumption was not associated with greater intakes of added sugars. Similar observations were seen in a US cohort using the NHANES database [18]. It is also important to note that the source of added sugars is an important consideration and that the adverse relationships may be specific to sugar-sweetened beverages and have not been shown for total sugars or yogurt [39]. Furthermore, the consumption of yogurt, whether flavoured or not, and with or without added sugar, has in fact been associated with health benefits, including reduced weight gain, reduced risk of developing T2D and other cardiometabolic diseases [40].

In addition to micronutrients, dairy foods such as yogurt are a source of high-quality protein due to their composition containing all nine essential amino acids that are readily digestible. In the present study, yogurt consumers had greater intakes of total dietary protein compared with non-consumers. Previous research has demonstrated the benefits that dairy proteins have on lowering glycaemic responses in healthy individuals [34,41,42,43]. Further, consumption of high-quality protein, such as from dairy, has been identified as an essential dietary intervention strategy to promote muscle health in the aging population [44]. With dietary guidance encouraging greater consumption of plant proteins, the market for plant alternatives to dairy has grown considerably since 2015. Currently, the sales of plant-based dairy alternatives account for 7.4% of the overall milk market share [45]. Therefore, it is reasonable to assume that a shift to a more plant-based eating pattern will include greater consumption of these products as a replacement for dairy, thereby compromising both protein and overall nutrient quality [46]. A recent study compared conventional dairy milk and plant-based beverages to their contribution to nutrient intake using CCHS-2015. The results showed that plain milk contributed to 12.5% of protein intake among dairy consumers, resulting in 6.5% greater protein than non-consumers, where plant alternatives to dairy contributed to only 1.4% of total protein intake. The authors concluded that plant alternatives to dairy are inferior to conventional dairy milk because they are lower in both protein quantity and quality [47]. More globally, it is important for individuals to obtain proteins from a variety of dietary sources, and public health messages should guide consumers to identify these foods. Recently, we observed that higher-quality and more nutrient-dense plant protein foods such as soy and pulses accounted for less than 15% daily protein intake among Canadians who were consuming at least 50% of their dietary protein from plant sources. Instead, refined grains were the majority protein contributors, accounting for a quarter of the daily protein intake [2,3,15]. Also, the EAT*Lancet* Commission recently updated their assessment on healthy, sustainable, and just food systems. In their report, while the authors mention that consumption of yogurt may result in lower weight gain, they also recommend no more than 1 serving of dairy per day and express concerns surrounding saturated fat content and increased levels of LDL cholesterol [48]. While the report is important in the context of human and planetary health, caution is warranted when failing to take into consideration food form and the complexity of the food matrix. The data in the present study are informative and point to the need for further research and communication on the role of dairy foods and the importance of dairy protein in healthy eating patterns and public health messaging.

While the results of our study highlight the meaningful contribution of yogurt consumption to diet quality, the data are from the CCHS-2015 and may not reflect current consumer trends. This supports a recent commentary that Canada urgently needs to implement updated and continuous dietary intake monitoring to enhance public health messaging and provide up-to-date formal recommendations [49]. Further research is needed to explore how yogurt consumption patterns interact with broader dietary trends and long-term health outcomes, particularly in the context of shifting dietary guidelines and evolving public health strategies.

### Strengths and Limitations

The present study provides the first estimate of the contribution of yogurt to the diet of Canadians as a snapshot of one-day food intakes across different age groups. We used CCHS 2015 Nutrition data, which are from the most recent national-level comprehensive nutrition and health database, providing estimates of mean food and nutrient intakes of Canadians. This report examined a wide range of nutrients, including macronutrients (protein, dietary fibre) and micronutrients (calcium, potassium, vitamin D), which are encouraged by Canadian dietary guidelines. Additionally, this study provides insights into the impact of yogurt consumption on added sugar intakes, which allows for a nuanced understanding of the nutritional role of yogurt that has not been before examined.

A limitation of this study is that it is based on a single-day recall (24 h), and, therefore, this may not be indicative of usual or habitual daily food intake. Additionally, we did not analyse the different types of yogurt products being consumed as the focus of the present study was to understand the contribution of yogurt to the nutrient quality of Canadian diets. However, with a growing market for different yogurt products, these secondary analyses would be informative. Nevertheless, our study supports a need to emphasize more frequent consumption of yogurt and other dairy products for the daily diet of all ages.

## 5. Conclusions

The results support a recommendation for a daily dietary pattern that includes yogurt. Being a yogurt consumer in this sample of Canadians resulted in higher daily intakes of several short-fall nutrients, such as protein, calcium, potassium, vitamin D, and dietary fibre. The findings highlight yogurt’s vital role in improving the nutrient status of Canadians, particularly for shortfall nutrients, such as protein, calcium, potassium, vitamin D, and dietary fibre. Given the evolving dietary landscape and the shift towards plant-based eating patterns, ensuring a place for nutrient-dense dairy foods like yogurt in daily dietary recommendations is essential. Future research should explore how sustained yogurt consumption contributes to long-term health outcomes, reinforcing its place in evidence-based dietary guidelines.

## Figures and Tables

**Table 1:** Reported age, sex, and household income across the groups by yogurt consumption.

	<1 g (n = 13,520)	1 g to 90 g (n = 1089)	>90 g to 115 g (n = 1244)	>115 g (n = 1455)
Age (mean ± SE)	42 ± 0.18	39 ± 1.13	38 ± 0.94	41 ± 0.81
Sex (n (%))				
Males	6848 (51%)	452 (42%)	490 (39%)	625 (43%)
Females	6672 (49%)	637 (58%)	754 (61%)	830 (57%)
Household income				
$0 to $39k	1378 (10%)	75 (7%)	72 (6%)	92 (6%)
$40k to $79k	6694 (50%)	511 (47%)	567 (46%)	653 (45%)
$80k to $119k	2750 (20%)	243 (22%)	290 (23%)	311 (21%)
>$120k	2698 (20%)	261 (24%)	315 (25%)	399 (27%)

**Table 2:** Nutrient content of all yogurts consumed on any given day (in yogurt consumers, n = 3788).

Nutrient	Mean ± SE
Energy (kcal)	97.67 ± 2.11
Protein (g)	6.34 ± 0.17
Carbohydrates (g)	13.04 ± 0.29
Fat (g)	2.30 ± 0.08
Total Sugars (g)	11.77 ± 0.26
Added Sugars (g)	6.63 ± 0.15
Sodium (mg)	56.99 ± 1.38
Calcium (mg)	161.50 ± 4.25
Vitamin D (mcg)	0.62 ± 0.03
Potassium (mg)	206.41 ± 5.05
Vitamin A (mcg)	22.79 ± 0.83
Vitamin B12 (mcg)	0.38 ± 0.01
Dietary Fibre (g)	0.14 ± 0.01

**Table 3:** Overall daily yogurt intake by DRI age/sex group in yogurt consumers (n = 3788).

DRI Age/Sex Groups	Yogurt Amount Consumed in Grams (Mean ± SE)
2 to 3 years old	119.80 ± 5.05
4 to 8 years old	126.53 ± 6.55
Males, 9 to 13 years old	124.00 ± 7.72
Females, 9 to 13 years old	115.13 ± 7.54
Males, 14 to 18 years old	113.12 ± 11.22
Females, 14 to 18 years old	140.47 ± 15.94
Males, 19 to 30 years old	127.48 ± 12.86
Females, 19 to 30 years old	168.42 ± 48.93
Males, 31 to 50 years old	133.09 ± 7.75
Females, 31 to 50 years old	130.85 ± 8.21
Males, 51 to 70 years old	145.36 ± 8.18
Females, 51 to 70 years old	129.99 ± 4.16
Males, >70 years old	121.71 ± 7.56
Females, >70 years old	118.13 ± 5.97

**Table 4:** Average overall nutrient intakes across the day by yogurt consumption across all ages.

All Ages	<1 g (n = 13,520)	1 g to 90 g (n = 1089)	>90 g to 115 g (n = 1244) ^1^	>115 g (n = 1455)
Energy (kcal)	1883.95 ± 14.88	1777.68 ± 50.22	1833.42 ± 37.94	1984.03 ± 32.26 **
Carbohydrates	123.42 ± 0.52 **	127.41 ± 1.56	127.54 ± 1.21	124.21 ± 1.67
Total Sugars	48.36 ± 0.38 ***	51.56 ± 1.05 **	54.97 ± 1.03	56.54 ± 1.01
Added Sugars	22.58 ± 0.36	20.55 ± 0.90	21.56 ± 0.75	22.85 ± 0.69
Fibre	9.34 ± 0.09 ***	10.65 ± 0.29	10.21 ± 0.22	10.09 ± 0.26
Protein	42.05 ± 0.26	41.86 ± 0.87	42.81 ± 0.53	45.24 ± 0.92 *
Total Fat	36.19 ± 0.17 **	35.30 ± 0.51	34.96 ± 0.44	35.90 ± 0.45
Saturated Fat	11.80 ± 0.08	11.84 ± 0.25	11.88 ± 0.18	12.44 ± 0.19 **
Sodium	1488.05 ± 9.63 *	1422.58 ± 29.24	1405.64 ± 38.15	1325.12 ± 22.95
Calcium	424.19 ± 3.47 ***	496.01 ± 12.87 *	537.97 ± 11.22	567.95 ± 9.19 *
Vitamin D	2.65 ± 0.05 ***	2.71 ± 0.12 **	3.05 ± 0.10	3.04 ± 0.12
Potassium	1455.89 ± 8.46 ***	1523.85 ± 32.59	1566.78 ± 18.74	1613.46 ± 21.36
Vitamin B12	2.19 ± 0.04	2.05 ± 0.26	2.53 ± 0.34	2.32 ± 0.09
Vitamin A	358.94 ± 5.67	364.69 ± 15.73	393.16 ± 94.80	392.08 ± 13.56

Mean value was significantly different from that of the >90 g to 115 g category: * *p* < 0.05, ** *p* < 0.01, *** *p* < 0.0001. ^1^ Group III was selected as the statistical comparator as it includes the commercial serving sizes and Health Canada’s reference amount of yogurt.

**Table 5:** Average nutrient intakes by yogurt consumption in children and adolescents (2 to 18 years).

Children and Adolescents (2 to 18 Years)	<1 g (n = 2915)	1 g to 90 g (n = 208)	>90 g to 115 g (n = 224) ^1^	>115 g (n = 248)
Energy (kcal)	1869.12 ± 22.15	1750.85 ± 48.24	1779.13 ± 50.43	1883.05 ± 43.44
Carbohydrates	135.02 ± 0.72	137.75 ± 1.59	136.19 ± 1.85	140.03 ± 1.68
Total Sugars	58.59 ± 0.63 **	61.98 ± 1.47	63.27 ± 1.68	69.85 ± 1.59 **
Added Sugars	25.89 ± 0.59	24.02 ± 1.25	23.31 ± 1.31	27.70 ± 0.96 ***
Fibre	8.61 ± 0.10	9.03 ± 0.28	9.17 ± 0.31	8.68 ± 0.22
Protein	38.99 ± 0.42 *	39.53 ± 0.70	41.05 ± 0.82	39.56 ± 0.65
Total Fat	35.06 ± 0.25	33.85 ± 0.63	34.00 ± 0.67	32.93 ± 0.65
Saturated Fat	12.34 ± 0.13	12.29 ± 0.32	12.31 ± 0.37	12.96 ± 0.32
Sodium	1426.95 ± 12.79 *	1383.17 ± 38.79	1341.81 ± 35.60	1338.03 ± 27.32
Calcium	499.79 ± 5.82 ***	578.96 ± 16.23	610.12 ± 17.85	659.64 ± 12.51 *
Vitamin D	3.07 ± 0.08 **	3.48 ± 0.22	3.75 ± 0.23	3.50 ± 0.13
Potassium	1321.69 ± 12.38 ***	1410.33 ± 28.48	1474.55 ± 35.49	1459.22 ± 28.61
Vitamin B12	2.16 ± 0.09	2.16 ± 0.08	2.35 ± 0.14	2.26 ± 0.08
Vitamin A	349.80 ± 10.36	346.38 ± 14.92	346.62 ± 19.45	359.92 ± 16.58

Mean value was significantly different from that of the >90 g to 115 g category: * *p* < 0.05, ** *p* < 0.01, *** *p* < 0.0001. ^1^ Group III was selected as the statistical comparator as it includes the commercial serving sizes and Health Canada’s reference amount of yogurt.

**Table 6:** Average nutrient intakes by yogurt consumption in adults (19 to 70 years).

Adults (19 to 70 Years)	<1 g (n = 7504)	1 g to 90 g (n = 480)	>90 g to 115 g (n = 627) ^1^	>115 g (n = 761)
Energy (kcal)	1931.70 ± 18.71	1830.13 ± 75.85	1886.55 ± 52.93	2053.44 ± 42.04
Carbohydrates	120.09 ± 0.70	121.98 ± 2.35	123.34 ± 1.69	118.48 ± 2.19
Total Sugars	45.66 ± 0.47 ***	46.16 ± 1.39 **	51.60 ± 1.40	52.11 ± 1.27
Added Sugars	21.92 ± 0.43	19.21 ± 1.29	21.14 ± 1.02	21.64 ± 0.93
Fibre	9.38 ± 0.13 ***	11.08 ± 0.43	10.42 ± 0.29	10.27 ± 0.35
Protein	42.96 ± 0.33	43.17 ± 1.34	44.12 ± 0.73	47.65 ± 1.32 *
Total Fat	36.58 ± 0.23	35.97 ± 0.76	35.84 ± 0.63	37.02 ± 0.60
Saturated Fat	11.64 ± 0.10	11.62 ± 0.36	11.88 ± 0.23	12.32 ± 0.25
Sodium	1501.28 ± 12.92	1435.91 ± 41.70	1443.96 ± 55.74	1302.65 ± 31.02 *
Calcium	405.65 ± 4.39 ***	452.78 ± 17.34 *	511.24 ± 16.20	541.43 ± 12.51
Vitamin D	2.47 ± 0.07 *	2.28 ± 0.14 **	2.79 ± 0.11	2.86 ± 0.16
Potassium	1474.79 ± 10.95 ***	1541.22 ± 49.75	1583.79 ± 24.12	1642.70 ± 28.21
Vitamin B12	2.17 ± 0.05	2.01 ± 0.41	2.71 ± 0.53	2.37 ± 0.13
Vitamin A	355.42 ± 7.49	362.76 ± 23.46	419.36 ± 146.12	403.65 ± 19.31

Mean value was significantly different from that of the >90 g to 115 g category: * *p* < 0.05, ** *p* < 0.01, *** *p* < 0.0001. ^1^ Group III was selected as the statistical comparator as it includes the commercial serving sizes and Health Canada’s reference amount of yogurt.

**Table 7:** Average nutrient intakes by yogurt consumption in elderly (>70 years).

Elderly (>70 Years)	<1 g (n = 1981)	1 g to 90 g (n = 132)	>90 g to 115 g (n = 168) ^1^	>115 g (n = 164)
Energy (kcal)	1600.57 ± 24.78	1538.99 ± 59.48	1679.69 ± 119.72	1741.45 ± 120.85
Carbohydrates	125.74 ± 1.15	132.24 ± 3.78	129.10 ± 3.18	128.81 ± 2.88
Total Sugars	48.90 ± 1.31	55.98 ± 2.71	52.91 ± 2.23	57.90 ± 1.70
Added Sugars	21.39 ± 1.02	19.28 ± 1.83	19.53 ± 1.24	20.72 ± 1.24
Fibre	10.28 ± 0.17 *	12.38 ± 0.56	11.59 ± 0.58	11.85 ± 0.60
Protein	41.25 ± 0.50	40.32 ± 1.50	40.02 ± 1.29	41.29 ± 1.24
Total Fat	35.49 ± 0.37 **	35.20 ± 1.25	32.57 ± 0.91	34.78 ± 1.07
Saturated Fat	11.93 ± 0.16 *	11.91 ± 0.67	10.83 ± 0.41	12.16 ± 0.56
Sodium	1503.24 ± 24.50	1447.80 ± 71.88	1354.81 ± 74.59	1444.42 ± 60.82
Calcium	419.32 ± 7.27 ***	532.38 ± 29.53	506.25 ± 23.61	551.21 ± 23.12
Vitamin D	3.12 ± 0.13	3.23 ± 0.38	2.80 ± 0.30	3.26 ± 0.23
Potassium	1554.87 ± 20.12 *	1720.64 ± 71.21	1699.78 ± 70.60	1741.27 ± 57.22
Vitamin B12	2.39 ± 0.11 *	2.06 ± 0.17	2.03 ± 0.13	2.08 ± 0.11
Vitamin A	396.72 ± 13.80	424.30 ± 29.07	365.45 ± 33.23	383.24 ± 25.36

Mean value was significantly different from that of the >90 g to 115 g category: * *p* < 0.05, ** *p* < 0.01, *** *p* < 0.0001. ^1^ Group III was selected as the statistical comparator as it includes the commercial serving sizes and Health Canada’s reference amount of yogurt.

## Data Availability

The data analysed in this manuscript belong to the public use microdata file (PUMF) from the Canadian Community Health Survey (CCHS) available here: https://www150.statcan.gc.ca/n1/en/catalogue/82M0013X (accessed on 4 February 2022–28 July 2025).

## Supplementary Materials

The following supporting information can be downloaded at: https://www.mdpi.com/article/10.3390/nu18101581/s1, Table S1: Nutrient intakes from yogurt only.
