# Supplementary material for: An Examination of the Effect of Yogurt Consumption on Nutrient Quality of the Diets of Canadians Across the Ages
Source: Nutrients. 2026 May 15;18(10):1581. doi: 10.3390/nu18101581 (PMC13209514; doi:10.3390/nu18101581)
Supplement: Supplementary file 1 [file nutrients-18-01581-s001.zip › nutrients-4267649-supplementary.pdf]

Supplement Table S1:

Table S1:Nutrient intakes from yogurt only:

OVERALL SAMPLE	<1g (n=13520)	1g to 90g (n=1089)	>90g to 115g (n=1244)	>115g (n=1455)
Energy (kcal)	0.00 ± 0.00	39.29 ± 2.11	79.79 ± 1.08	153.70 ± 3.93
Carbohydrates	0.00 ± 0.00	5.05 ± 0.24	11.09 ± 0.19	20.30 ± 0.53
Total Sugars	0.00 ± 0.00	4.49 ± 0.21	10.13 ± 0.19	18.27 ± 0.48
Added Sugars	0.00 ± 0.00	2.53 ± 0.12	5.71 ± 0.11	10.29 ± 0.28
Fibre	0.00 ± 0.00	0.05 ± 0.01	0.15 ± 0.02	0.19 ± 0.03
Protein	0.00 ± 0.00	2.57 ± 0.12	4.92 ± 0.11	10.19 ± 0.36
Total Fat	0.00 ± 0.00	1.01 ± 0.13	1.81 ± 0.07	3.62 ± 0.18
Saturated Fat	0.00 ± 0.00	0.65 ± 0.08	1.14 ± 0.04	2.29 ± 0.12
Sodium	0.00 ± 0.00	22.97 ± 0.94	44.66 ± 0.48	91.21 ± 2.94
Calcium	0.00 ± 0.00	66.89 ± 3.31	127.45 ± 2.17	256.50 ± 9.58
Vitamin D	0.00 ± 0.00	0.25 ± 0.02	0.56 ± 0.02	0.93 ± 0.06
Potassium	0.00 ± 0.00	83.80 ± 3.31	157.00 ± 0.92	333.88 ± 10.76
Vitamin B12	0.00 ± 0.00	0.16 ± 0.01	0.31 ± 0.00	0.59 ± 0.02
Vitamin A	0.00 ± 0.00	9.86 ± 0.64	17.99 ± 0.93	35.92 ± 1.84

Supplementary Materials: Table S1:Nutrient intakes from yogurt only.
